# Longitudinal amyloid PET changes by cerebrospinal fluid amyloid and tau profiles in individuals with normal cognition: Role of *APOE* ε4

**DOI:** 10.1177/13872877261453099

**Published:** 2026-05-30

**Authors:** Marianna Rizzo, Julie E. Oomens, Willemijn J. Jansen, Pieter Jelle Visser, Stephanie J. B. Vos, on behalf of the AMYPAD consortium

**Affiliations:** 1 Department of Psychiatry and Neuropsychology, Alzheimer Centrum Limburg, Mental Health and Neuroscience Research Institute, 82246 Maastricht University, Maastricht, Netherlands; 2 601873 Amsterdam Neuroscience, Neurodegeneration, Amsterdam, The Netherlands; 3 Alzheimer Center Amsterdam, Neurology, 685848 Vrije Universiteit Amsterdam, Amsterdam UMC location VUmc, Amsterdam, The Netherlands; 4 Department of Neurobiology, Care Sciences and Society, Division of Neurogeriatrics, 27106 Karolinska Institutet, Stockholm, Sweden

**Keywords:** Alzheimer's disease, amyloid, amyloid-β peptides, Apolipoprotein E4, cerebrospinal fluid, cohort studies, longitudinal studies, positron emission tomography, tau proteins

## Abstract

**Background:**

Identifying factors associated with early progression of cortical amyloid deposition is crucial for understanding Alzheimer's disease (AD) pathophysiology, yet these remain unclear.

**Objective:**

To investigate the associations of AD cerebrospinal fluid (CSF) biomarkers with early longitudinal amyloid deposition on positron emission tomography (PET), and the impact of *APOE* ε4 carriership.

**Methods:**

We selected cognitively unimpaired participants with baseline data on CSF amyloid and p-tau181, *APOE* ε4 carriership, and ≥ 2 consecutive amyloid PET assessments from the AMYPAD-PNHS cohort. Linear mixed models were used to investigate the associations of combinations of amyloid abnormality (A+) and tau abnormality (T+) in CSF (A+T+, A+T-, A-T+, A-T-), without and with stratification for *APOE* ε4 carriership, with longitudinal global cortical amyloid deposition on PET, measured in Centiloids.

**Results:**

We included 329 individuals (mean age 63.5 [SD 6.1], 58% female, 49% *APOE* ε4 carriers). Longitudinally, CSF profiles with abnormal amyloid levels (A+T+, A+T-) showed greater increases in cortical amyloid deposition than profiles with normal amyloid levels (A-T+, A-T-), irrespective of CSF tau abnormality (all p < 0.001). Only for CSF profiles with normal amyloid levels, longitudinal amyloid deposition depended on *APOE* ε4 carriership, with both A-T+ and A-T- showing increased deposition among *APOE* ε4 carriers (carriers versus non-carriers: A-T+ p = 0.006, A-T- p = 0.016).

**Conclusions:**

In cognitively unimpaired individuals, longitudinal increase in cortical amyloid deposition was mainly associated with abnormal baseline CSF amyloid rather than tau. *APOE* ε4 carriership was associated with increased longitudinal cortical amyloid deposition only in individuals with normal baseline CSF amyloid. These findings inform early AD pathological progression and trial design.

## Introduction

Alzheimer's disease (AD) affects more than 40 million people worldwide and is a top challenge for healthcare due to the lack of effective treatments.^1^ One of the earliest pathophysiological events in AD is the accumulation of amyloid pathology, which starts up to 20 years before the onset of clinical symptoms, and is thought to trigger downstream tau deposition, neurodegeneration, and eventually cognitive decline. Knowledge on the early-stage predictors of longitudinal amyloid deposition is crucial for understanding AD pathophysiology, yet these remain poorly understood.

Current well-established modalities to assess AD biomarkers (i.e., amyloid and tau) *in vivo* are a positron emission tomography (PET) scan or cerebrospinal fluid (CSF) collection. Previous work indicates that CSF detects earlier AD-related amyloid or tau processes, whereas PET quantifies the later deposition of amyloid or tau pathology in the brain.^2–7^ Classification of cognitively unimpaired individuals based on amyloid status (A) and tau status (T) in CSF can therefore provide useful prognostic information in early AD stages.^8,9^ Although CSF AT profiles are widely used in research and clinical contexts, the longitudinal cortical amyloid deposition trajectories of these profiles in cognitively unimpaired individuals are still largely unexplored. This gap can be partly attributable to the scarcity of cohorts with longitudinal amyloid PET follow-up in cognitively unimpaired individuals. To our knowledge, only one study has examined this in persons without dementia and has shown that, compared to individuals with a A-T- baseline CSF profile, those with a A+T- or A+T+ profile displayed increased cortical amyloid deposition longitudinally, whereas those with a A-T+profile did not.^10^ Yet, the impact of *APOE* ε4 on these trajectories remains unknown. The *APOE* ε4 allele is a well-established genetic AD risk factor known to be overall associated with increased amyloid deposition and earlier AD onset,^11–13^ which must be considered when assessing longitudinal amyloid trajectories. To enable appropriate patient stratification in clinic, research, and trial settings, it is important to characterize its effects separately. Knowledge on how early longitudinal amyloid deposition is predicted by key AD biomarkers and *APOE* ε4 is also important as the field progresses towards treatments aimed to prevent amyloid deposition into plaques.

Therefore, the aims of this study were (1) to investigate, in cognitively unimpaired individuals, the associations of baseline CSF AT biomarker profiles with longitudinal deposition of cortical amyloid on PET, and (2) to assess the effect of *APOE* ε4 on these associations. We hypothesized an increased longitudinal cortical amyloid deposition for CSF AT profiles at more advanced biomarker stages (A+T+ > A+T- > A-T+ > A-T-), and a more pronounced deposition in *APOE* ε4 carriers than non-carriers.

## Methods

### Design and cohort

This retrospective cohort study used data from the AMYloid Imaging to Prevent Alzheimer's Disease (AMYPAD) Prognostic and Natural History Study (PNHS, dataset version 202306). AMYPAD-PNHS is a prospective, multi-center, longitudinal cohort study which recruited participants between 2016 and 2022. The study design and recruitment procedures are described in detail elsewhere.^14,15^ Briefly, AMYPAD-PNHS aimed to assess the value of quantitative amyloid PET imaging to predict AD progression. To this end, participant data from the centers included in AMYPAD-PNHS were complemented with ≥1 amyloid PET scans. Recruitment aimed at individuals without a dementia diagnosis aged ≥50 from the full AD risk spectrum (i.e., individuals with negative, gray zone, and positive AD biomarkers). After at least one year, participants were invited for a follow-up PET scan, subject to local site capacity.

Included cohorts were: ALzheimer's and Families + (ALFA+, N = 147)^16^; European Prevention of Alzheimer's Dementia Longitudinal Cohort Study (EPAD-LCS, N = 91)^17^; European Medical Information Framework for Alzheimer's disease—PreclinAD study (EMIF-AD 60++, N = 81)^18^; Longitudinal Cognitive Impairment and Dementia Study (DELCODE, N = 6)^19^; and Fundació ACE Healthy Brain Initiative (FACEHBI, N = 4).^20^ An overview of participating cohorts is also available at https://amypad.eu/project/amypad-pnhs/. ALFA + recruited participants via a population-based enrolment at the Barcelonaβeta Brain Research Center (Barcelona), EPAD-LCS via existing European research cohorts (e.g., population-based, prevention trials) or clinical/routine care cohorts (e.g., memory clinic or primary care), EMIF-AD 60++ via the Netherlands Twin Register (Amsterdam), DELCODE via memory clinics or public advertisement in Germany, FACEHBI via referral by general practitioners at the Fundació ACE memory clinic (Barcelona) or via an OpenHouse Initiative.

### Standard protocol approvals, registrations, and patient consents

Cohorts obtained approval of protocol and relevant documentation by the local Ethics Committee or Institutional Review Board. All cohort studies were performed in accordance with the Declaration of Helsinki. Participants provided written informed consent before enrollment. AMYPAD-PNHS is registered at www.clinicaltrialsregister.eu with the EudraCT Number: 2018-002277-22.

### Participants

We included all AMYPAD-PNHS cognitively unimpaired individuals with baseline and at least one follow-up amyloid PET assessments, and baseline data on CSF amyloid-β (Aβ)_42/40_ or Aβ_42_, CSF p-tau181, and *APOE* ε4 carriership. A baseline Clinical Dementia Rating (CDR)^21^ global score of 0 was used to define cognitively unimpaired status, as previously applied in several studies.^22–26^ CSF was collected close in time to baseline PET scan and at most within 1 year (Supplemental Table 1).

### CSF AT profiles and APOE ε4 carriership

CSF samples were collected following established standard procedures at each center, where CSF Aβ_42_ or Aβ_42/40_ and CSF p-tau181 levels were measured. For ALFA + and EPAD-LCS (238 participants), Aβ_42_ and p-tau181 were quantified using the Roche Elecsys assay at the Clinical Neurochemistry Laboratory at the University of Gothenburg, Sweden.^27–29^ For EMIF-AD 60++ (81 participants), Aβ_42/40_ and p-tau181 were quantified using the ADx Neurosciences/Euroimmun.^18^ For DELCODE (6 participants), Aβ_42_ was quantified using V-PLEX Aβ Peptide Panel 1, and p-tau181 using ELISA immunoassay (INNOTEST Fujirebio Europe, Goteborg, Sweden), which was also used to quantify Aβ_42_ and p-tau181 in FACEHBI (4 participants). Established cutoffs provided by cohorts^19,30–34^ (Supplemental Table 2) were used to classify participants according to their baseline CSF Aβ_42_ or Aβ_42/40_ (A) and p-tau181 (T) normal (-) or abnormal (+) status into four AT profiles: A-T-, A-T+, A+T-, and A+T+ . Participants were subsequently classified as *APOE* ε4 carriers if they carried at least one ε4 allele.

### Amyloid PET assessment

Amyloid PET acquisition and processing are described in more detail elsewhere.^14,15,35^ Briefly, [^18^F]florbetaben (NeuraCeq) and [^18^F]flutemetamol (Vizamyl) radiotracers were used. Centiloid (CL) quantification of PET images was performed to allow pooled analyses across tracers. In the Centiloid scale, 0 represents absence of amyloid in young controls, and 100 represents the amyloid burden of typical mild-to-moderate AD dementia.^36^ PET images were intensity normalized using the whole cerebellum as reference, as per standard Centiloid protocol (https://www.gaain.org/centiloid-project). A measure of global cortical amyloid deposition was then computed by averaging Centiloid values throughout the cortex. Additionally, for this study, a measure of deposition in an early-AD-stage region of interest (ROI) was obtained by averaging Centiloid values in the precuneus and cingulate cortex (anterior, middle, and posterior), as a variable sensitive to early AD-related amyloid increases.^37,38^

### Statistical analysis

Baseline characteristics were compared across the four CSF AT profiles using ANOVA for continuous variables and chi-squared for categorical variables. Linear mixed models with random intercepts and slopes for participant and cohort were used to evaluate the longitudinal cortical amyloid deposition on PET for each CSF AT biomarker profile and the impact of *APOE* ε4 carriership. In the main analysis, the four baseline CSF AT profiles were used as predictor, and amyloid deposition in the global cortical region as outcome (regression equation shown at Supplemental Table 3). In a sensitivity analysis, amyloid deposition in the early-AD-stage ROI was used as outcome. Next, to assess the impact of *APOE* ε4, each CSF AT profile was stratified into *APOE* ε4 carriers and non-carriers (eight groups in total). These groups were used as categorical predictor in a model including amyloid deposition in the global cortical region as outcome (regression equation shown at Supplemental Table 3). All models were adjusted for age at baseline (i.e., at the first PET scan) and biological sex (male/female), as older age and female sex are known risk factors for amyloid deposition in AD.^39^ In sensitivity analyses, we additionally adjusted for the time interval between baseline PET scan and CSF collection. We chose linear model fits over nonlinear fits based on the inspection of the annual rate of change in global cortical amyloid deposition, as previously applied (Supplemental Figure 1).^40–42^ This choice aligns with evidence indicating an overt plateau in amyloid accumulation over time only after the asymptomatic stage and the amyloid positivity threshold in AD course.^43^ Statistical analyses were performed using R and SPSS version 27.0.1.0 (Armonk, NY), and a two-sided p < .05 was used to define statistical significance. In a sensitivity analysis, p-values were corrected for multiple comparisons using the Holm-Bonferroni method, controlling the family-wise error rate for the number of comparisons performed in each model. Data were analyzed between 2023 and 2026. The current study followed the Strengthening the Reporting of Observational Studies in Epidemiology (STROBE) reporting guidelines.

## Results

### Sample characteristics

We included 329 out of 3368 individuals of the AMYPAD-PNHS dataset. Baseline characteristics are shown in Table 1. Participants had a mean baseline age of 63.5 (SD 6.1) years, 191 (58%) were female, and 169 (49%) were *APOE* ε4 carriers. Mean years of formal education completed were 14.4 (SD 3.8) and mean Mini-Mental State Examination (MMSE) score was 29.2 (SD 0.93). All individuals had a single amyloid PET follow-up, except three who had two follow-ups. Average PET follow-up time was 3.2 (SD 1.0, range 1–6) years. 169 (51%) individuals were classified as A-T-, 69 (21%) as A-T+, 59 (18%) as A+T-, and 32 (10%) as A+T+ in CSF at baseline. CSF abnormal-tau groups (A+T+, A-T+) were on average older than normal-tau groups. CSF abnormal-amyloid groups (A+T+, A+T-) were more often *APOE* ε4 carriers than CSF normal-amyloid groups. Sex distribution, education, and MMSE were similar across AT groups. The average PET follow-up years were 3.0 (SD 1.0, range 1–5) for the A-T- group, 3.7 (SD 1.0, range 1–6) for the A-T+ group, 2.9 (SD 1.0, range 1–4) for the A+T- group, and 3.3 (SD 1.0, range 1–5) for the A+T+ group.

**Table 1.** Baseline sample characteristics.

	A-T- (n = 169)	A-T+ (n = 69)	A+T- (n = 59)	A+T+ (n = 32)	Total sample (n = 329)
Age	62.9 (5.6)^b,d^	65.4 (5.9)^a,c^	61.2 (6.4)^b,d^	66.9 (6.0)^a,c^	63.5 (6.1)
Female sex (%)	102 (60)	37 (54)	32 (54)	20 (63)	191 (58)
Education years	14.6 (3.5)	15.2 (4.6)	14.6 (3.3)	14.2 (4.3)	14.7 (3.8)
*APOE* ε4 carriers (%)	60 (36)^c,d^	30 (43)^c,d^	47 (80)^a,b^	23 (72)^a,b^	169 (51)
*APOE* ε4 heterozygotes (%)	51 (30)^c,d^	30 (43)	36 (61)^a^	18 (56)^a^	135 (41)
MMSE score	29.3 (0.9)	29.2 (1.0)	29.3 (0.9)	29.0 (1.0)	29.2 (0.9)
CSF amyloid z-score	0.18 (0.88)^c,d^	−0.16 (1.48)^c,d^	−1.55 (0.46)^a,b,d^	−3.02 (2.26)^a,b,c^	−0.51 (1.57)
CSF p-tau181 z-score	−0.13 (0.69)^b,d^	1.55 (1.45)^a,c,d^	−0.32 (0.72)^b,d^	3.38 (3.08)^a,b,c^	0.53 (1.74)

The table shows key baseline characteristics of the total sample and of each cerebrospinal fluid (CSF) amyloid (A) and tau (T) profile. “+”: positive status; “-“: negative status; MMSE: Mini-Mental State Examination; *APOE* ε4: Apolipoprotein E ε4. Values represent mean (SD) for continuous variables or participant number (%) for dichotomous variables. MMSE score was available for 294 participants. Z-scores for CSF Aβ_42_ or Aβ_42/40_ and p-tau181 were computed using a reference population with low amyloid levels on PET (CL < 12), normal MMSE (>27), normal CDR (CDR global = 0), age ≤70, and without *APOE* ε4 carriership. Variables were compared across the AT profiles using ANOVA for continuous variables and chi-square for categorical variables. When the overall difference between profiles was significant, post-hoc testing between groups was conducted with t-tests for continuous variables and pairwise proportion test for categorical variables. Superscript letters (a-d) indicate significant (p < 0.05) unadjusted group differences compared to: a) A-T-, b) A-T+, c) A+T-, d) A+T+.

### Cortical amyloid deposition on PET by CSF AT profiles

**Figure 1.**
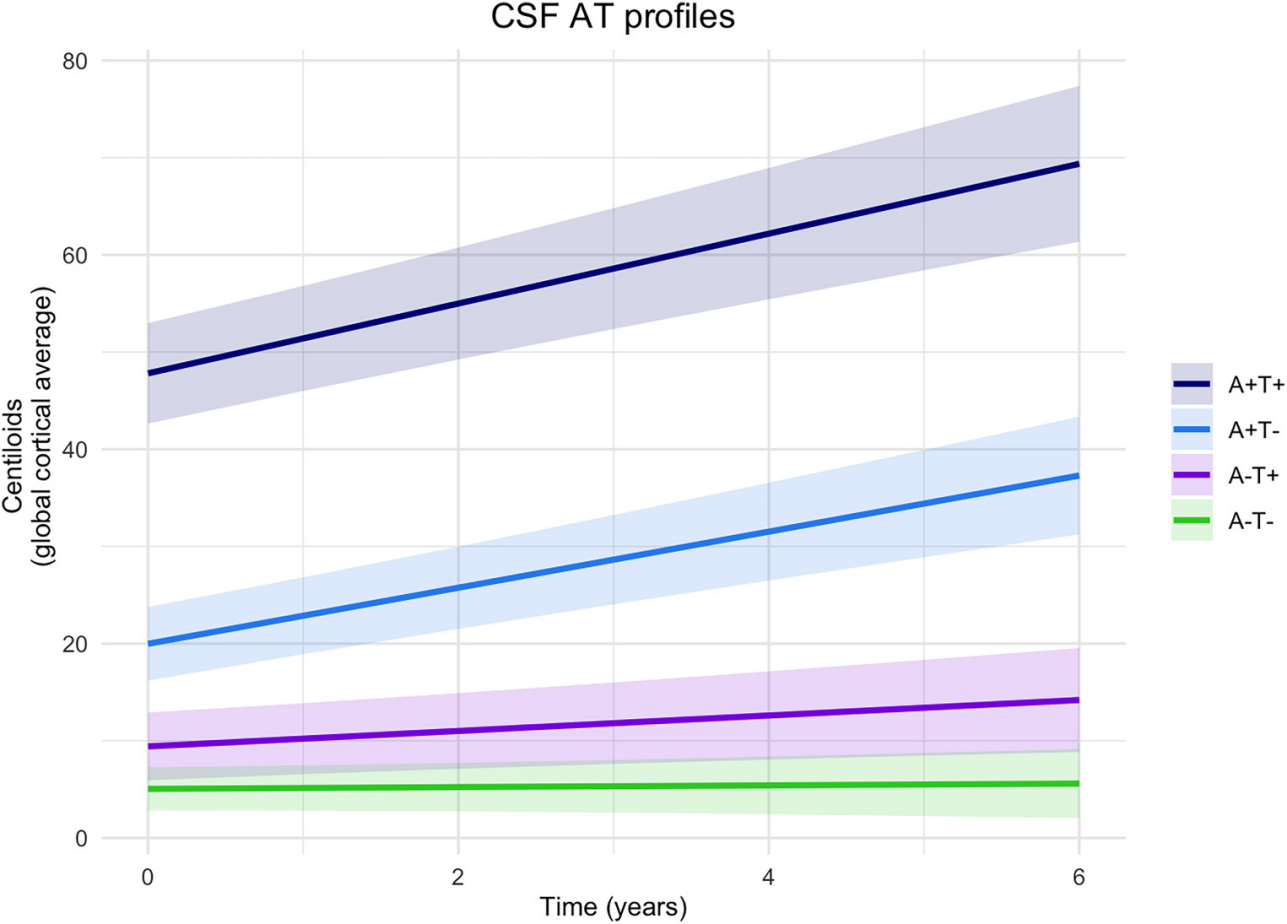
Longitudinal change in cortical amyloid deposition by CSF AT group. The graph shows the predicted relationship between baseline CSF AT profiles and longitudinal amyloid deposition in the global cortical region measured in Centiloids. Lines are linear mixed modelling slopes, with shades depicting the 95% confidence intervals. A+: abnormal amyloid; A-: normal amyloid; T+: abnormal tau; T-: normal tau, in cerebrospinal fluid (CSF) at baseline.

Baseline deposition and longitudinal change in global cortical amyloid on PET by CSF AT profiles are shown in Table 2 and Figure 1. Baseline global cortical amyloid deposition levels were different between all CSF AT groups. Levels were lowest in A-T- (M = 5.1 [2.8,7.3]; p = 0.039 compared to A-T+; p < 0.001 compared to A+T- and to A+T+), higher in A-T+ (M = 9.4 [5.9,12.9], p < 0.001 compared to A+T-), followed by A+T- (M = 20.0 [16.2,23.8], p < 0.001 compared to A+T+), and highest in A+T+ (M = 47.8 [42.6,53.0]).

**Table 2.** Baseline and longitudinal change in global cortical amyloid deposition by CSF AT group.

	A-T-	A-T+	A+T-	A+T+
N	169	69	59	32
Baseline	5.1 (2.8, 7.3) ^b,c,d^	9.4 (5.9, 12.9) ^a,c,d^	20.0 (16.2, 23.8) ^a,b,d^	47.8 (42.6, 53.0) ^a,b,c^
Slope	0.1 (−0.3, 0.5) ^c,d^	**0.8** **(0.2, 1.4)** ^c,d^	**2.8** **(2.2, 3.6)** ^a,b^	**3.6** **(2.7, 4.5)** ^a,b^

The table shows baseline and slope estimates (95% confidence interval) of the linear mixed model analysis, adjusted for baseline age and sex. The slope estimates indicate Centiloid change per year. A+: abnormal amyloid; A-: normal amyloid; T+: abnormal tau; T-: normal tau, in cerebrospinal fluid at baseline. Significant slope estimates are in bold (p < 0.05). Superscript letters (a-d) indicate significant (p < 0.05) group differences compared to: a) A-T-, b) A-T+, c) A+T-, d) A+T+ .

Longitudinally, A+T+, A+T-, and A-T+ showed an increase in cortical amyloid deposition (A+T+ β = 3.6 [2.7,4.5], p < 0.001; A+T- β = 2.9 [2.2,3.6], p < 0.001; A-T+ β = 0.8 [0.2–1.4], p = 0.006), whereas A-T- did not display any change (β = 0.1 [-0.3,0.5], p = 0.661). Abnormal-amyloid groups (A+T+, A+T-) showed greater increases in longitudinal amyloid deposition than normal-amyloid groups (A-T+, A-T-), irrespective of tau status (A+T+ versus A-T+ β = 2.8 [1.8,3.8], p < 0.001; A+T+ versus A-T- β = 3.5 [2.5,4.5], p < 0.001; A+T- versus A-T+ β = 2.1 [1.2,3.0], p < 0.001; A+T- versus A-T- β = 2.8 [2.0,3.6], p < 0.001). No differences in longitudinal amyloid deposition were observed among abnormal-amyloid groups (A+T+ versus A+T- β = 0.7 [-0.4,1.8], p = 0.212), or among normal-amyloid groups (A-T+ versus A-T- β = 0.7 [0.0,1.4], p = 0.046). Non-covariate adjusted estimates are reported in Supplemental Table 4.

Results did not change after correction for multiple comparisons. Results also remained similar when additionally adjusting for the time interval between baseline PET scan and CSF collection (Supplemental Table 5). When using the early-AD-stage ROI as outcome measure, we observed higher baseline cortical amyloid levels and steeper increases for all groups compared to when using the global cortical region, but overall results remained similar (Supplemental Table 6).

### Impact of APOE ε4 carriership

**Figure 2.**
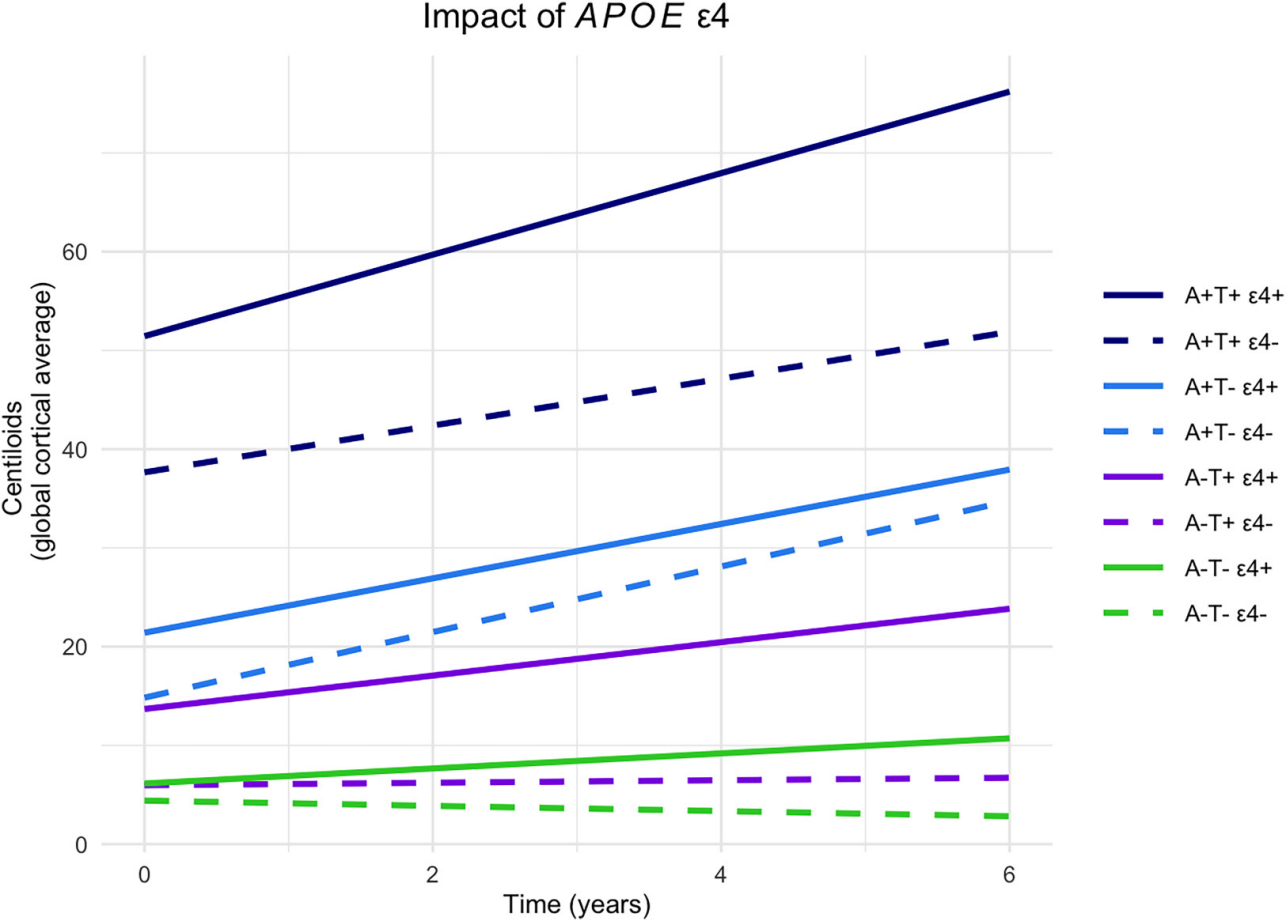
Impact of *APOE* ε4 carriership on longitudinal change in cortical amyloid deposition by CSF AT group. The graph shows the predicted relationship between baseline CSF AT profiles stratified by *APOE* ε4 carriership and longitudinal amyloid deposition in the global cortical region measured in Centiloids. Lines are linear mixed modelling slopes. 95% confidence intervals were omitted for better visualization. A+: abnormal amyloid; A-: normal amyloid; T+: abnormal tau; T-: normal tau, in cerebrospinal fluid (CSF) at baseline; ε4+: *APOE* ε4 carriers, ε4-: *APOE* ε4 non-carriers.

Results stratified by *APOE* ε4 carriership are shown in Figure 2 and Table 3. At baseline, *APOE* ε4 carriers had higher cortical amyloid deposition than non-carriers only in abnormal-tau groups (A+T+ carriers versus non-carriers M = 13.8 [2.6,24.9], p = 0.016; A-T+ carriers versus non-carriers M = 7.7 [0.8,14.6], p = 0.029). When comparing AT groups, unlike in the overall analysis, *APOE* ε4 non-carriers A+T- showed similar baseline amyloid deposition to A-T+ (M = 8.9 [-0.6,18.4], p = 0.067), and A-T+ to A-T- (M = 1.6 [-3.8,6.9], p = 0.565), whereas in *APOE* ε4 carriers baseline amyloid deposition remained different between all AT groups.

**Table 3.** Baseline and longitudinal change in global cortical amyloid deposition by CSF AT group in *APOE* ε4 carriers and non-carriers.

		A-T-	A-T+	A+T-	A+T+
*APOE* ε4 carriers	N	60	30	47	23
	Baseline	6.1 (2.2, 9.8)^b,c,d^	13.7 (8.5, 18.5) ^a,c,d,2^	21.4 (17.2, 25.6)^a,b,d^	51.5 (45.5, 57.4)^a,b,c,4^
	Slope	**0.8** **(0.9, 1.4)** ^c,d,1^	**1.7** **(0.9, 2.5)** ^d,2^	**2.8** **(2.0, 3.5)** ^a,d^	**4.1** **(3.1, 5.2)** ^a,b,c^
*APOE* ε4 non-carriers	N	109	39	12	9
	Baseline	4.4 (1.7, 7.1)^c,d^	6.0 (1.3, 10.6) ^d,2^	14.8 (6.6, 23.0)^a,d^	37.7 (28.1, 47.2)^a,b,c,4^
	Slope	−0.3 (−0.8, 0.2)^c,d,1^	0.1 (−0.6, 0.9)^c,d,2^	**3.3** **(1.8, 4.8)** ^a,b^	**2.4** **(0.9, 3.9)** ^a,b^

The table shows baseline and slope estimates (95% confidence interval) of the linear mixed model analysis, adjusted for baseline age and sex. The slope estimates indicate Centiloid change per year. A+: abnormal amyloid; A-: normal amyloid; T+: abnormal tau; T-: normal tau, in cerebrospinal fluid at baseline; *APOE* ε4: apolipoprotein E ε4. Significant slope estimates are in bold (p < 0.05). Superscript letters (a-d) indicate, within *APOE* ε4 carriers and within non-carriers, significant (p < 0.05) group differences compared to: a) A-T-, b) A-T+, c) A+T-, d) A+T+ . Superscript numbers (1-4) denote group comparisons between *APOE* ε4 carriers and non-carriers and indicate significant (p < 0.05) group differences compared to: 1) A-T- with opposite *APOE* ε4 carriership status, 2) A-T+ with opposite *APOE* ε4 carriership status, 4) A+T+ with opposite *APOE* ε4 carriership status.

Longitudinally, in normal-amyloid groups (A-T+ and A-T-), only *APOE* ε4 carriers showed an increase in cortical amyloid (A-T+ β = 1.7 [0.8,2.5], p < 0.001; A-T- β = 0.8 [0.1,1.4], p = 0.027), while non-carriers did not (A-T+ β = 0.1 [-0.6,0.9], p = 0.727; A-T+ carriers versus non-carriers β = 1.6 [0.5,2.7], p = 0.006; A-T- β = -0.3 [-0.8,0.2], p = 0.293; A-T- carriers versus non-carriers β = 1.0 [0.2,1.9], p = 0.016). When comparing AT profles, unlike in the overall analysis, only in *APOE* ε4 carriers A-T+ showed a similar longitudinal increase to A+T- (A-T+ carriers versus A+T- carriers β = 1.1 [-0.1,2.2], p = 0.067). Conversely, in abnormal-amyloid groups, both carriers and non-carriers showed a similar longitudinal increase in amyloid deposition (A+T+ carriers β = 4.1 [3.1,5.2], p < 0.001; A+T+ non-carriers β = 2.4 [0.8,3.9], p = 0.002; A+T+ carriers versus non-carriers β = 1.8 [-0.1,3.6], p = 0.061; A+T- carriers β = 2.8 [2.0,3.5], p < 0.001; A+T- non-carriers β = 3.3 [1.8, 4.8], p < 0.001; A+T- carriers versus non-carriers β = -0.6 [-2.3,1.1], p = 0.513). When comparing AT profiles, unlike in the overall analysis, in *APOE* ε4 carriers A+T+ showed a steeper increase in longitudinal amyloid deposition than A+T- (A+T+ carriers versus A+T- carriers β = 1.4 [0.1,2.7], p = 0.036).

After correction for multiple comparisons, a few findings were attenuated, described hereafter. At baseline, within abnormal-tau groups, the higher cortical amyloid deposition in *APOE* ε4 carriers compared to non-carriers was observed at trend level (A+T+ carriers versus non-carriers p_adj_ = 0.062; A-T+ carriers versus non-carriers p_adj_ = 0.086). Similarly, longitudinally, the increase in cortical amyloid shown by A-T- *APOE* ε4 carriers was observed as a trend (p_adj_ = 0.082); however, its comparison with A-T- non-carriers remained significant (p_adj_ = 0.049). Finally, within *APOE* ε4 carriers, A+T+ showed a similar longitudinal increase in amyloid deposition as A+T- (p_adj_ = 0.107). All results remained similar when additionally adjusting for the time interval between baseline PET scan and CSF collection (Supplemental Table 7).

## Discussion

Our study aimed to investigate the early changes in cortical amyloid deposition on PET shown by baseline CSF amyloid and tau biomarker profiles, and the impact of *APOE* ε4 carriership on these changes. Our main finding is that participants with CSF abnormal amyloid profiles at baseline (i.e., A+T+ and A+T-) deposited cortical amyloid over an average of 3 years largely independently of *APOE* ε4 carriership. Conversely, participants with CSF normal amyloid profiles (i.e., A-T+ and A-T-) showed increased cortical amyloid deposition longitudinally among *APOE* ε4 carriers compared to non-carriers.

Baseline cortical amyloid deposition was higher in more advanced CSF AT stages (A+T+ > A+T- > A-T+ > A-T-). While a higher cortical amyloid load is expected with CSF amyloid positivity, the additional association with CSF tau positivity suggests that soluble tau CSF levels may increase as a reaction to amyloid plaques.^44^ Longitudinally, CSF abnormal amyloid profiles showed greater increases in cortical amyloid deposition than normal amyloid profiles. Interestingly, this was mainly independent of tau status. This highlights the importance of CSF amyloid, rather than tau, in predicting the subsequent rate of deposition of amyloid into plaques.

Analyses stratified for *APOE* ε4 carriership showed that individuals with CSF normal amyloid but abnormal tau levels who were *APOE* ε4 carriers (A-T+ ε4+) had a slightly higher baseline cortical amyloid deposition and a higher longitudinal increase in cortical amyloid deposition than those who were not *APOE* ε4 carriers (A-T+ ε4-). Interestingly, the Centiloid levels showed by A-T+ *APOE* ε4 carriers were within the known range of emerging amyloid pathology (10–30 CL) since baseline (≈14 CL) and reached levels consistent with established pathology on visual read (≈21 CL) at year 4, and histology (≈24 CL) at year 6.^45^ Additionally, the longitudinal amyloid deposition shown by this group (A-T+ ε4+) was comparable to that shown by the A+T- group. Conversely, the Centiloid levels of A-T+ *APOE* ε4 non-carriers remained below amyloid positivity thresholds (10 CL).^45^ A-T+ is often referred to as suspected non-Alzheimer's disease pathophysiology (SNAP). A SNAP profile has previously been found to be indistinguishable from A-T- with respect to longitudinal cortical amyloid deposition in individuals both without cognitive impairment^46^ and without dementia,^10^ strengthening the conclusion that SNAP does not pertain to the AD continuum. However, neuropathology studies suggest that SNAP could be early AD.^47^ Our results suggest that *APOE* ε4 carriership may identify individuals with a CSF A-T+ profile at risk for amyloid deposition. The importance of *APOE* genotype in accounting for the genetic differences between SNAP and A+T+ has already been implicated in an earlier study on MCI individuals.^48^ Typically, SNAP is associated with a lower prevalence of *APOE* ε4 carriership, which suggests that different neurodegeneration mechanisms than AD are at play.^49,50^ In our study, the prevalence of *APOE* ε4 carriership was also lower compared to groups within the AD continuum (A+T- and A+T+), yet it remained notably higher (43%) than previously reported (26%).^47,51^ This could potentially be explained by the partial inclusion in the current study of individuals with subjective cognitive decline from clinical settings. Follow-up population studies on A-T+ *APOE* ε4 carriers are needed to validate the observed amyloid PET progression with long-term assessments and evaluate whether a longitudinal increase in amyloid deposition is accompanied by cognitive decline.

The group with normal amyloid and tau CSF biomarkers (A-T-) also displayed increased longitudinal cortical amyloid deposition among *APOE* ε4 carriers compared to non-carriers. Although the Centiloid levels were relatively low and only exceeded thresholds for emerging amyloid pathology (i.e., 10 CL)^45^ at year 6, this finding highlights the importance of selecting *APOE* ε4 non-carriers as controls in studies evaluating biomarker and clinical trajectories. Indeed, some A-T- *APOE* ε4 carriers may still develop amyloid pathology, possibly combined with other AD risk factors. Identifying such additional contributors is increasingly important as the field advances towards interventions that target amyloid prior to deposition into fibrillary plaques.

Finally, among individuals with abnormal amyloid, *APOE* ε4 carriers showed a slightly higher baseline plaque load than non-carriers only in individuals with concurrent tau abnormality (A+T+). Yet, Centiloid levels of both A+T+ carriers and non-carriers met criteria for established amyloid pathology (>30 CL), and longitudinally, *APOE* ε4 carriership did not appreciably influence the rate of cortical amyloid deposition. Altogether, our results suggest that in cognitively unimpaired individuals, *APOE* ε4 carriership is associated with an increased rate of cortical amyloid deposition when individuals still have normal amyloid levels. These findings are in line with an earlier study on individuals without dementia showing that *APOE* ε4 was associated with increased cortical amyloid deposition rates only in individuals who had normal, but not abnormal, amyloid levels on PET at baseline.^52^ Moreover, this aligns with the several studies indicating that *APOE* ε4 shifts the onset of amyloid deposition to an earlier time, and suggests that once established, amyloid deposition progresses at a consistent rate independent of *APOE* ε4 status.^11,53,54^

Mechanistically, our results suggest that *APOE* ε4 may have primarily effects on amyloid seeding, rather than propagation.^55,56^ Several mechanisms may underlie this phenomenon. *APOE* ε4 may impair amyloid clearance, thereby increasing the extracellular availability of amyloid and its proneness to aggregate.^55,57^ *APOE* ε4-associated microglia dysfunction may impair the microglial-mediated phagocytosis of nascent amyloid aggregates, thereby allowing their persistence and deposition.^56,57^ Furthermore, *APOE* ε4's structural conformation may stabilize early amyloid forms, favoring their accumulation.^57,58^ *APOE* ε4-related alterations in lipid metabolism may additionally promote amyloid misfolding and initial aggregation at lipid membranes.^55,58^

Other studies in cognitively unimpaired individuals have however found that *APOE* ε4 carriership was related to a faster amyloid deposition rate in the precuneus in individuals with high baseline amyloid PET load,^59^ or after accounting for baseline amyloid PET levels both in individuals with abnormal and normal amyloid.^12^ Our study expands existing literature on *APOE* ε4 roles by accounting for factors which have previously not been examined. We considered the concurrent role of baseline tau, rather than focusing solely on baseline amyloid, in longitudinal amyloid deposition. In addition, by using baseline CSF, rather than PET, amyloid and tau measures, we characterized the impact of *APOE* ε4 on earlier AD changes, rather than later-stage ones. Our results have implications for *APOE*-targeting therapies^60^ and suggest that these may be most effective in prevention trials when initiated before substantial amyloid aggregation, aligning with the conclusions of a recent study.^61^

Key strengths of our study are a sample of cognitively unimpaired individuals with multimodal biomarker measures (CSF and PET), and longitudinal amyloid PET data with up to 6 years of follow-up. Additionally, the leverage of commonly available CSF biomarkers (Aβ_42_ or Aβ_42/40_ and p-tau181) to estimate future amyloid plaque load enhanced the clinical relevance of the study. The use of the recently validated Centiloid metric for amyloid PET contributed to data standardization across cohorts. However, there are also limitations to our study. First, the known dose effects of the *APOE* ε4 allele could not be investigated due to insufficient sample size. Secondly, measures of tau via the recently validated CSF p-tau217 (more strongly associated with tau PET uptake than CSF p-tau181)^62^ were unavailable. Aβ_42_ levels were not referenced to Aβ_40_ in ≈3/4 of our sample due to its unavailability, making our amyloid measure potentially susceptible to some non-disease related variability.^63^ Moreover, it must be noted that after stratification by *APOE* ε4 status, amyloid-positive groups of *APOE* ε4 non-carriers had relatively small sample sizes, potentially affecting the robustness of the results. This participant distribution is however expected to be similar in other cohorts, as amyloid positivity is commonly related to *APOE* ε4 carriership. Additionally, we did not use a three-way interaction analysis between CSF AT profiles, *APOE* ε4, and time to evaluate *APOE* ε4 carriership's impact on the profile trajectories, as such an analysis may produce non-robust estimates in our relatively small sample and may not detect effects that exist only for certain profiles. Given the strong a-priori evidence to examine *APOE* ε4 as an effect modifier, our stratified analyses are clinically informative, but they remain potentially vulnerable to small-group instability while increasing the number of comparisons. Another limitation is that our study sample was predominantly white and therefore not representative of the general population. Finally, the relationship between changes in continuous amyloid deposition on PET and shifts in CSF AT status over time could not be examined due to the limited availability of longitudinal CSF data. Future research should address these limitations and expand our study by including a larger participant group with a wider range of outcomes, such as tau PET, diagnostic information, and cognitive measures.

In summary, our study showed that in cognitively normal individuals, both CSF amyloid and tau abnormality were associated with a higher baseline amyloid plaque load, but CSF amyloid abnormality, rather than tau, was related to an increased future rate of amyloid plaque deposition. *APOE* ε4 carriership was associated with the initial deposition of cortical amyloid plaques only in individuals with normal baseline CSF amyloid levels, especially when combined with elevated CSF tau. These findings give important insights into AD pathology progression and may help the design of AD clinical trials.

## Supplemental Material

sj-docx-1-alz-10.1177_13872877261453099 - Supplemental material for Longitudinal amyloid PET changes by cerebrospinal fluid amyloid and tau profiles in individuals with normal cognition: Role of *APOE* ε4 Supplemental material, sj-docx-1-alz-10.1177_13872877261453099 for Longitudinal amyloid PET changes by cerebrospinal fluid amyloid and tau profiles in individuals with normal cognition: Role of *APOE* ε4 by Marianna Rizzo, Julie E. Oomens, Willemijn J. Jansen, Pieter Jelle Visser, Stephanie J. B. Vos and in Journal of Alzheimer's Disease
