# Supplementary material for: Longitudinal amyloid PET changes by cerebrospinal fluid amyloid and tau profiles in individuals with normal cognition: Role of APOE ε4
Source: J Alzheimers Dis. 2026 May 30;112(2):875–85. doi: 10.1177/13872877261453099 (PMC13334065; doi:10.1177/13872877261453099)
Supplement: sj-docx-1-alz-10.1177_13872877261453099 - Supplemental material for Longitudinal amyloid PET changes by cerebrospinal fluid amyloid and tau profiles in individuals with normal cognition: Role of APOE ε4 [file sj-docx-1-alz-10.1177_13872877261453099.docx]

**Supplemental Material**

**Longitudinal amyloid PET changes by cerebrospinal fluid amyloid and tau profiles in individuals with normal cognition: Role of *APOE* ε4**

## Supplemental Table 1. Time interval between CSF collection and PET scan.

| **Time interval** | **Number of participants** |
| --- | --- |
| 7-12 months before the baseline PET scan | 44 |
| ± 6 months of the baseline PET scan | 285 |

The table shows the number of participants for each time interval between CSF collection and baseline PET scan.

## Supplemental Table 2. Quantification of CSF markers by each cohort.

| **Cohort** | **Cases** | **Assay** | **Marker** | **Cutoff** |
| --- | --- | --- | --- | --- |
| ALFA+ | 147 | Roche Elecsys | Aβ_42_ | <1098 pg/mL |
|  |  |  | p-tau181 | >19.2 pg/mL |
| EPAD-LCS | 91 | Roche Elecsys | Aβ_42_ | <1000 pg/mL |
|  |  |  | p-tau181 | >27 pg/mL |
| EMIF-AD 60++ | 81 | ADx Neurosciences/ Euroimmun | Aβ_42/40_ | <0.066 pg/mL |
|  |  |  | p-tau181 | >58 pg/mL |
| DELCODE | 6 | V-PLEX Aβ Peptide Panel 1 | Aβ_42_ | <496 pg/mL |
|  |  | ELISA immunoassay INNOTEST | p-tau181 | >57 pg/mL |
| FACEHBI | 4 | ELISA immunoassay INNOTEST | Aβ_42_ | <676 pg/mL |
|  |  |  | p-tau181 | >58 pg/mL |

The table shows marker, kit, and cutoff used for the quantification of CSF Aβ and p-tau per each cohort.

## Supplemental Table 3. Regression equations.

| **Analysis** | **Regression equation** |
| --- | --- |
| Cortical amyloid deposition by CSF AT profiles | Global_region_centiloids = β0 + β1*(baseline_age) + β2*(sex) + β3*(baseline_CSF_AT_profile) + β4*(PET_years) + β5*(baseline_CSF_AT_profile*PET_years) + b0_subject_cohort + b1_subject_cohort*(PET_years) + ε |
| Impact of *APOE* ε4 carriership on cortical amyloid deposition by CSF AT profiles | Global_region_centiloids = β0 + β1*(baseline_age) + β2*(sex) + β3*(baseline_CSF_AT_profile_APOE4) + β4*(PET_years) + β5*( baseline_CSF_AT_profile_APOE4 *PET_years) + b0_subject_cohort + b1_subject_cohort*(PET_years) + ε |

The table shows the regression equations used to quantify the baseline and longitudinal cortical amyloid deposition of the CSF AT profiles, and the impact of *APOE* ε4 carriership.

## Supplemental Table 4. Baseline and longitudinal change in global cortical amyloid deposition by CSF AT group, unadjusted model.

|  | A-T- | A-T+ | A+T- | A+T+ |
| --- | --- | --- | --- | --- |
| N | 169 | 69 | 59 | 32 |
| Baseline | 4.9  (2.6, 7.2) ^b,c,d^ | 10.8  (7.2, 14.3) ^a,c,d^ | 18.5  (14.6, 22.3) ^a,b,d^ | 50.4  (45.1, 55.6) ^a,b,c^ |
| Slope | 0.1  (-0.3, 0.5) ^c,d^ | **0.8**  **(0.2, 1.4)** ^c,d^ | **2.9**  **(2.2, 3.6)** ^a,b^ | **3.6**  **(2.7, 4.5)** ^a,b^ |

The table shows baseline and slope estimates (95% confidence interval) of the linear mixed model analysis, not adjusted for baseline age and sex. The slope estimates indicate Centiloid change per year. A+: abnormal amyloid; A-: normal amyloid; T+: abnormal tau; T-: normal tau, in cerebrospinal fluid at baseline. Significant slope estimates are in bold (p<0.05). Group comparisons p<0.05 compared to: a) A-T-, b) A-T+, c) A+T-, d) A+T+.

## Supplemental Table 5. Baseline and longitudinal change in global cortical amyloid deposition by CSF AT group, additionally adjusted for time between baseline PET and CSF collection.

|  | A-T- | A-T+ | A+T- | A+T+ |
| --- | --- | --- | --- | --- |
| N | 169 | 69 | 59 | 32 |
| Baseline | 5.1  (2.8, 7.3) ^b,c,d^ | 9.4  (5.9, 12.9) ^a,c,d^ | 20.0  (16.2, 23.8) ^a,b,d^ | 47.8  (42.6, 53.0) ^a,b,c^ |
| Slope | 0.1  (-0.3, 0.5) ^c,d^ | **0.8**  **(0.2, 1.4)** ^c,d^ | **2.9**  **(2.2, 3.6)** ^a,b^ | **3.6**  **(2.7, 4.5)** ^a,b^ |

The table shows baseline and slope estimates (95% confidence interval) of the linear mixed model analysis, adjusted for baseline age, sex, and time interval in months between baseline PET scan and CSF collection. The slope estimates indicate Centiloid change per year. A+: abnormal amyloid; A-: normal amyloid; T+: abnormal tau; T-: normal tau, in cerebrospinal fluid at baseline. Significant slope estimates are in bold (p<0.05). Group comparisons p<0.05 compared to: a) A-T-, b) A-T+, c) A+T-, d) A+T+.

## Supplemental Table 6. Baseline and longitudinal change in cortical amyloid deposition in early-AD-stage ROI by CSF AT group.

|  | A-T- | A-T+ | A+T- | A+T+ |
| --- | --- | --- | --- | --- |
| N | 169 | 69 | 59 | 32 |
| Baseline | 20.3  (18.0, 22.7) ^b,c,d^ | 25.9  (22.2, 29.5) ^a,c,d^ | 35.1  (31.1, 39.1) ^a,b,d^ | 66.5  (61.1, 71.9) ^a,b,c^ |
| Slope | 0.3  (-0.2, 0.7) ^c,d^ | **1.0**  **(0.4, 1.7)** ^c,d^ | **3.3**  **(2.5, 4.0)** ^a,b^ | **3.9**  **(3.0, 4.9)** ^a,b^ |

The table shows baseline and slope estimates (95% confidence interval) of the linear mixed model analysis, adjusted for baseline age and sex. The slope estimates indicate Centiloid change per year. A+: abnormal amyloid; A-: normal amyloid; T+: abnormal tau; T-: normal tau, in cerebrospinal fluid at baseline. Significant slope estimates are in bold (p<0.05). Group comparisons p<0.05 compared to: a) A-T-, b) A-T+, c) A+T-, d) A+T+.

## Supplemental Table 7. Baseline and longitudinal change in global cortical amyloid deposition by CSF AT group in *APOE* ε4 carriers and non-carriers, additionally adjusted for time between baseline PET and CSF collection.

|  | | A-T- | A-T+ | A+T- | A+T+ |
| --- | --- | --- | --- | --- | --- |
| *APOE* ε4 carriers | N | 60 | 30 | 47 | 23 |
|  | Baseline | 6.1  (2.4, 9.8) ^b,c,d^ | 13.7  (8.5, 18.9) ^a,c,d,2^ | 21.4  (17.2, 25.6) ^a,b,d^ | 51.4  (45.5, 57.4) ^a,b,c,4^ |
|  | Slope | **0.8**  **(0.9, 1.4)** ^c,d,1^ | **1.7**  **(0.8, 2.5)** ^d,2^ | **2.8**  **(2.0, 3.5)** ^a,d^ | **4.1**  **(3.1, 5.2)** ^a,b,c^ |
| *APOE* ε4 non-carriers | N | 109 | 39 | 12 | 9 |
|  | Baseline | 4.4  (1.7, 7.2) ^c,d^ | 6.0  (1.3, 10.6) ^d,2^ | 14.8  (6.6, 23.0) ^a,d^ | 37.6  (28.0, 47.2) ^a,b,c,4^ |
|  | Slope | -0.3  (-0.8, 0.2) ^c,d,1^ | 0.1  (-0.6, 0.9) ^c,d,2^ | **3.3**  **(1.8, 4.8)** ^a,b^ | **2.4**  **(0.9, 3.9)** ^a,b^ |

The table shows baseline and slope estimates (95% confidence interval) of the linear mixed model analysis, adjusted for baseline age, sex, and time interval in months between baseline PET scan and CSF collection. The slope estimates indicate Centiloid change per year. A+: abnormal amyloid; A-: normal amyloid; T+: abnormal tau; T-: normal tau, in cerebrospinal fluid at baseline; *APOE* ε4: apolipoprotein E ε4. Significant slope estimates are in bold (p<0.05). Group comparisons within *APOE* ε4 carriers and non-carriers p<0.05 compared to: A) A-T-, B) A-T+, C) A+T-, D) A+T+. Group comparisons between *APOE* ε4 carriers and non-carriers p<0.05 compared to: 1) A-T- with opposite *APOE* ε4 carriership status; 2) A-T+ with opposite *APOE* ε4 carriership status; 4) A+T+ with opposite *APOE* ε4 carriership status.


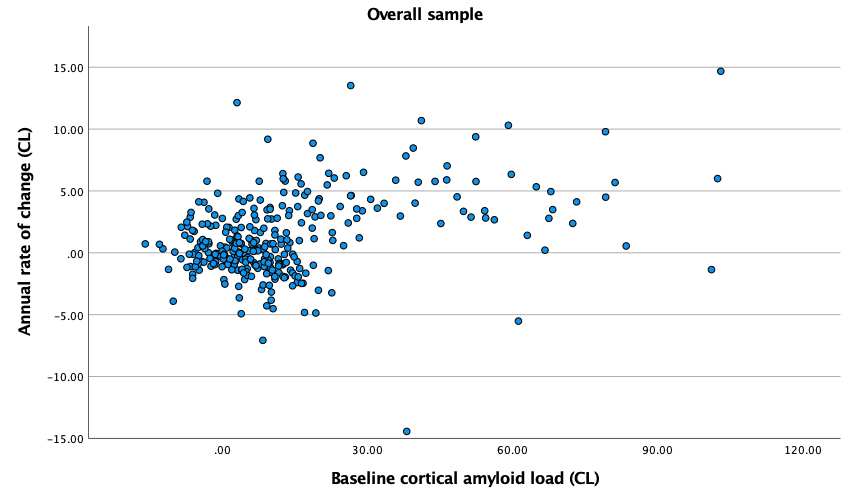


A

B

## Supplemental Figure 1. Uncorrected annual rate of change in amyloid deposition.

The graphs plot the uncorrected annual rate of change in global cortical amyloid deposition (CL) against baseline global cortical amyloid deposition (CL) in the overall group (section A) and in the CSF AT profiles (section B). The uncorrected annual rate of change was obtained using the following formula: *(Follow-up scan – Baseline scan) / Follow-up duration in years.*
